# An opportunity for clinical pharmacology trained physicians to improve patient drug safety: A retrospective analysis of adverse drug reactions in teenagers

**DOI:** 10.12688/f1000research.14970.2

**Published:** 2018-08-14

**Authors:** Andy R. Eugene, Beata Eugene

**Affiliations:** 1 Department of Pharmacogenomics, Bernard J. Dunn School of Pharmacy, Inova Center for Personalized Health, Shenandoah University, Fairfax, VA, 22031, USA; 2 Neurophysiology Unit, Department of Psychiatry, Medical University of Lublin, Aleje Racławickie 1, 20-059 Lublin, Poland; 3 Marie-Curie Sklodowska University, Lublin, Poland

**Keywords:** adverse drug reactions, pharmacogenomics, psychiatry, precision medicine, pharmacogenomics, consult, mental health, teenagers

## Abstract

**Background:** Adverse drug reactions (ADRs) are a major cause of hospital admissions, prolonged hospital stays, morbidity, and drug-related mortality. In this study, we sought to identify the most frequently reported medications and associated side effects in adolescent-aged patients in an effort to prioritize clinical pharmacology consultation efforts for hospitals seeking to improve patient safety.

**Methods: **Quarterly reported data were obtained from the United States Food and Drug Administration Adverse Events Reporting System (FAERS) from the third quarter of 2014 and ending in the third quarter of 2017. We then used the GeneCards database to map the pharmacogenomic biomarkers associated with the most reported FAERS drugs. Data homogenization and statistics analysis were all conducted in R for statistical programming.

**Results: **We identified risperidone (10.64%) as the compound with the most reported ADRs from all reported cases. Males represented 90.1% of reported risperidone cases with gynecomastia being the most reported ADR. Ibuprofen OR=188 (95% CI, 105.00 – 335.00) and quetiapine fumarate OR=116 (95% CI, 48.40 – 278.00) were associated with the highest odds of completed suicide in teenagers. Ondansetron hydrochloride OR=7.12 (95% CI, 1.59 – 31.9) resulted in the highest odds of pneumothorax. Lastly, olanzapine (8.96%) represented the compound with the most reported drug-drug interactions cases, while valproic acid OR=221 (95% CI, 93.900 – 522.00) was associated with the highest odds of drug-drug interactions.

**Conclusion: **Despite any data limitations, physicians prescribing risperidone in males should be aware of the high rates of adverse drug events and an alternative psychotropic should be considered in male patients. Further, patients with a history of pneumothorax or genetically predisposed to pneumothorax should be considered for an alternative antiemetic to ondansetron hydrochloride, due to increased odds associated with the drug and adverse event.

## Introduction

When considering the aims of precision medicine, which has the underlying theme of maximizing therapeutic efficacy while minimizing adverse drug reactions, all physician and surgeon specialists serve an integral role in achieving the overall goal of this national endeavor ( Manolio, 2016; Rasmussen-Torvik *et al.*, 2014; Weinshilboum & Wang, 2017). Within medical specialties, clinical pharmacologists are vital for providing pharmacogenomics consultations to patients, to other specialists, and in academic medicine to support the widespread implementation of pharmacogenomics and personalized medicine ( Borobia *et al.*, 2018; Moore, 2001; van der Wouden *et al.*, 2017). Further, there is a growing need to provide more genomic medicine training modules to physicians in non-academic medical centers and rural clinics to support patient care decisions that address pharmacogenomics ( McCauley *et al.*, 2017). Within the United States, the American Board of Clinical Pharmacology (ABCP) accredits institutions that train clinical pharmacologists who consult on patient cases of drug-gene interactions (i.e. pharmacogenomics), drug-drug interactions (DDIs), drug-drug-gene interactions, toxicology cases, and the use of pharmacometric tools that provide Bayesian dosing support for therapeutic drug monitoring (TDM) ( Aronson, 2012; Lewis & Nierenberg, 2007). However, when implementing hospital-based clinical pharmacology consultation units, aside from the established drug-gene guidelines, what is a reasonable approach for hospital pharmacologists to prioritize medications that are associated with the most reported adverse drug reactions that will improve hospital safety outcomes?

It is well-known that thousands of adverse drug reactions resulting in hospitalizations, increased lengths of hospital stay, and complications in patient management occur every year ( Montané *et al.*, 2018; Schmiedl *et al.*, 2014). However, an approach that systematically addresses the top medications associated with the most reported adverse drug events, leading to a prioritization method for hospital pharmacologists to improve medication safety, is lacking ( Davies *et al.*, 2009; Shepherd *et al.*, 2012). Several healthcare institutions in the United States have well established physician clinical pharmacology training programs, and integrate experiences into daily patient care, medical education, and research ( Lewis & Nierenberg, 2007). The University of Chicago Hospital, in conjunction with the Indiana Institute for Personalized Medicine at the Indiana University, offers a clinical pharmacology consultation service that conducts pharmacogenomic consults to low-income patients and provide thorough documentation of its process in a 2016 publication ( Eadon *et al.*, 2016). Other ABCP-accredited institutions (e.g. Mayo Clinic, Johns Hopkins Hospital, Baylor College of Medicine, Cincinnati Children’s Hospital, and more) are training and leading the U.S. with various pharmacogenomics implementation strategies into routine patient care (see ABCP training programs).

In European nations and other countries with national health systems, the intrinsic goal of keeping all healthcare-related costs to a minimum and hospital re-admissions rates to a low, while still maintaining high-quality patient care, medical doctors specializing in clinical pharmacology who provide personalize medicine services are the norm ( Borobia *et al.*, 2018; Janković *et al.*, 2016; Zagorodnikova Goryachkina *et al.*, 2015). Contrastingly, the multi-payer model currently within U.S. hospitals, often preclude hospitals from absorbing the cost of a clinical pharmacologists who would translate pharmacogenetics guidelines into daily patient care.

It is important to note that hospitals with clinical pharmacology training programs are often ranked among the top ranked by U.S. news and world reports, even though clinical pharmacology is not one of the specialties being assessed for survival, patient safety, other care-related outcomes, and expert opinion ( Harder *et al.*, 2017). The service and commitment to the use of precision dosing in patient care, research, clinical pharmacology education, and pharmacogenomics implementation at these hospitals provide an overall compelling story. One of the most well recognized hospitals, globally, is the Karolinska Institutet in Stockholm, Sweden, due its awarding of the Nobel Prize in Physiology or Medicine. A recent article by the Karolinska Institutet discusses how the 50 year jubilee was recently celebrated in recognition of the establishment of their hospital’s Department of Clinical Pharmacology ( Eichelbaum *et al.*, 2018).

In the recent jubilee article, the Karolinska Institutet’s clinical pharmacologists detail the various established responsibilities of their clinical pharmacology services, which function as a division within the department of laboratory medicine today, and how they addressed this vital unmet clinical need within their medical center ( Eichelbaum *et al.*, 2018). In the U.S., a National Provider Identifier taxonomy code for clinical pharmacology is well established as 208U00000X; however, hospitals and state medical boards have not worked with state legislative officials to create a bill enacting medical licensure (e.g. independent, collaborative, or institutional) specifically for medical school graduates who enter directly into clinical pharmacology training. Yet, adverse drug reactions continue to affect outcomes and patient safety metrics each year ( Burkhart *et al.*, 2015; Montané *et al.*, 2018).

It is important to realize that collaborative practice agreement laws between licensed physicians and pharmacists, physician assistants, and nurses are already in existence, but remains un-addressed for medical school graduates who choose only to specialize and train in clinical pharmacology. Therefore, if nothing is done, national implementation of precision medicine remains a challenge, due to not having enough trained medical doctors who focus on implementing pharmacogenomics into patient care and contribute to pharmacogenomics education ( McCauley *et al.*, 2017; Rosenman *et al.*, 2017).

With this information as a background, the primary aim of this article is to determine the most frequently reported drugs and associated adverse drug reactions that are found within the FDA Adverse Events Reporting System (FAERS) that will aid in prioritizing efforts for clinical pharmacology consultation services. To do so, we will access publically available FAERS data and report reporting frequencies and reporting odd-ratios of cases in an adolescent patient age group to avoid polypharmacy, albeit not exclusively in all cases.

## Methods

### Data

The United States Food and Drug Administration’s (FDA) Adverse Events Reporting System (FAERS) quarterly reports were downloaded, with dates ranging from the third quarter of 2014 to the third quarter of 2017. The ‘primaryid’ column, which represents a unique number of case sequence identifiers and manufacturer version number, were systematically linked as the primary field to other individual data files. Prior to our retrospective data analysis, we removed duplicate cases and selected reports classified from the adolescent (12 – 17 years-old) age group alone. A source of bias in the FAERS quarterly files may be underreporting of drugs in particular people groups due to language. Institutional Review Board approval was not required due to the FAERS data being public de-identified patient cases.

The following are the data tables for each quarter (i.e. Q1-Q4) of the year (i.e. yy in the files): patient demographic and administrative information (DEMOyyQ1-Q4), drug/biologic information (DRUGyyQ1-Q4), the Medical Dictionary for Regulatory Activities (MedDRA) terms of reported adverse events (REACyyQ1-Q4), patient outcomes (OUTCyyQ1-Q4), report sources (RPSRyyQ1-Q4), drug therapy start dates and end dates (THERyyQ1-Q4), and finally the MedDRA terms coded for the clinical indications (INDIyyQ1-Q4). Links to data used can be found in Table 1.

**Table 1.** Data used in this study are referenced from the U.S. Food and Drug Administration’s quarterly reported adverse events reporting system data.

Reporting time	Quarterly file name	File web address
**July - September** **2017**	FAERS_ASCII_2017q3 (ZIP - 42.1MB)	https://www.fda.gov/downloads/Drugs/GuidanceComplianceRegulatoryInformation/ Surveillance/UCM590948.zip
**April - June 2017**	FAERS_ASCII_2017q2 (ZIP - 41.3MB)	https://www.fda.gov/downloads/Drugs/GuidanceComplianceRegulatoryInformation/ Surveillance/UCM578242.zip
**January - March** **2017**	FAERS_ASCII_2017q1 (ZIP - 42.6MB)	https://www.fda.gov/downloads/Drugs/GuidanceComplianceRegulatoryInformation/ Surveillance/AdverseDrugEffects/UCM562290.zip
**October -** **December 2016**	FAERS ASCII 2016q4 (ZIP - 39.2MB)	https://www.fda.gov/downloads/Drugs/GuidanceComplianceRegulatoryInformation/ Surveillance/UCM546946.zip
**July - September** **2016**	FAERS ASCII 2016q3 (ZIP - 40.6MB)	https://www.fda.gov/downloads/Drugs/GuidanceComplianceRegulatoryInformation/ Surveillance/AdverseDrugEffects/UCM534900.zip
**April - June 2016**	FAERS ASCII 2016q2 (ZIP - 43.7MB)	https://www.fda.gov/downloads/Drugs/GuidanceComplianceRegulatoryInformation/ Surveillance/UCM521951.zip
**January - March** **2016**	FAERS ASCII 2016q1 (ZIP - 45.4MB)	https://www.fda.gov/downloads/Drugs/GuidanceComplianceRegulatoryInformation/ Surveillance/UCM509489.zip
**October -** **December 2015**	FAERS ASCII 2015q4 (ZIP - 39.7MB)	https://www.fda.gov/downloads/Drugs/GuidanceComplianceRegulatoryInformation/ Surveillance/AdverseDrugEffects/UCM492340.zip
**July - September** **2015**	FAERS ASCII 2015q3 (ZIP - 44.7MB)	https://www.fda.gov/downloads/Drugs/GuidanceComplianceRegulatoryInformation/ Surveillance/UCM477190.zip

### Mapping of Drug-Gene Targets

The primary and secondary molecular target mappings of the top FAERS reported drugs were obtained from the compounds listed in the GeneCards database. ( Stelzer *et al.*, 2016; Weizmann Institute of Science, 2016). GeneCards uses an Inferred Functionality Score that provides an objective number that indicates the knowledge level about the functionality of human genes relative to the drug queried in the database by comparing the drug with all possible genes. The final results are ranked according to a relevance score and reported in the results section of this article. In our analysis, we mapped the top ten genes using the GeneCards methodology, as has been previously reported ( Stelzer *et al*., 2016; Weizmann Institute of Science, 2016).

### Statistics

All data homogenization and statistics were computed using R for Statistical Computing (version 3.3.2, Vienna, Austria) programming software ( R Core Team, 2015). The top 15 indications, adverse drug reactions, and drugs are reported for the adolescent age group. The frequency tables were calculated based on: (number of drugs or adverse effect events) / (number of patient records) = drug or adverse events frequency. The reporting odds-ratios (OR), that scans across the medications under test, for a particular reported adverse drug event, are calculated using “Diarrhoea” as the control preferred term while “Hyperglycaemia”, “Pneumothorax”, and “Completed suicide” preferred terms were used for cases. The glm() function and binomial statistical family in R were used to conduct the logistic regression analysis. The following expression details the equation for the reporting odds-ratio: OR = (drug-of-interest group with adverse event / control-drug group with adverse event) / (control-drug group with adverse event / control-drug group without an adverse event). The ‘control-drug group with adverse event’ is set to “Diarrhoea” due to that being the most reported adverse drug event and is most commonly reported in FDA reports in the adolescent age population of patients. Morever, as mentioned, the cases (e.g. drug-of-interest group with adverse event) were set to “Hyperglycaemia”, “Pneumothorax”, and “Completed suicide.” Odds-ratios are reported as: odds-ratio, lower-95% confidence-interval (CI), upper-95% CI, and p-value. A p-value of less than 0.05 was considered to be statistically significant.

## Results

**Figure 1.**
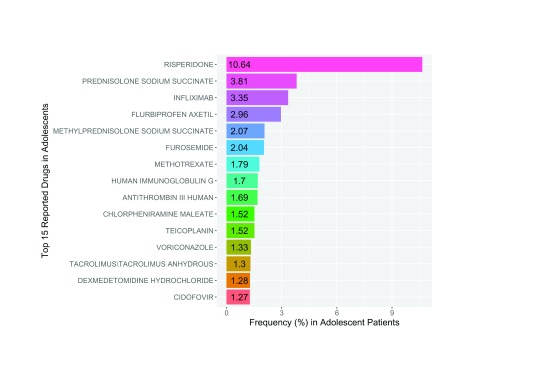
Frequencies for the top 15 reported drugs in adolescent patient records identified in the FDA Adverse Events Reporting system ranging from the 3 ^rd^ quarter of 2014 to the 3 ^rd^ quarter of 2017.

The study included a total of 6,141 unique cases (male=2938, female=3021, undefined=184) for adolescent-aged patient records, out of a total of 22,784 unique pediatric cases. The compound with the most reported adverse drug reactions was risperidone (n=788) representing 10.64% of all reported cases. We found that of the reported risperidone cases, 90.1% (male=710, female=77, undefined=1) were reported in men alone. The top 10 reported genes associated with risperidone are (GeneCards score): *DRD2* (dopamine receptor D2; 25.88), *PRL* (Prolactin; 25.59), *HTR2A* (5-hydroxytryptamine receptor 2A; 21.38), *CYP2D6* (cytochrome P450 Family 2 Subfamily D Member 6; 20.21), *HTR2C* (14.32), *ABCB1* (ATP binding cassette subfamily B member 1; 13.63), *BDNF* (brain derived neurotropic factor; 11.93), *DRD3* (11.79), *HTR1A* (11.35), and *CYP3A5* (11.03). Figure 1 illustrates the reporting frequencies of the top 15 reported drugs in adolescents.

**Figure 2.**
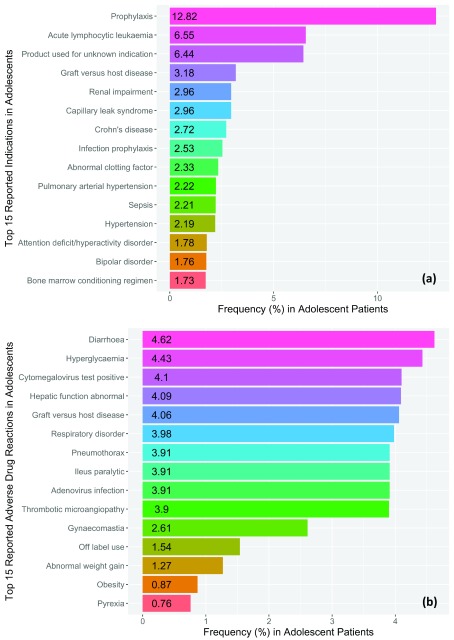
Frequencies for the top 15 reported ( **a**) clinical indications and ( **b**) adverse drug reactions (ADRs) in adolescent patient records identified in the FDA Adverse Events Reporting system ranging from the 3 ^rd^ quarter of 2014 to the 3 ^rd^ quarter of 2017.

The most commonly reported clinical indication was prophylaxis (12.82%), followed by acute lymphocytic leukemia (6.55%), and product used for unknown indication (6.44%). Figure 2a illustrates the reporting frequencies of the top fifteen reported clinical indications in the adolescent patient records from our study. The most reported adverse drug reaction was diarrhea (n=110, male=55, female=53, undefined=2) which represented 4.62% of the all of the reported cases. Following diarrhea, hyperglycemia (n=45, male=35, female=10) was the second most reported adverse drug event representing 4.43% of all reported cases. Figure 2b illustrates the reporting frequencies of the top fifteen reported adverse drug reactions for all adolescent cases.

We conducted logistic regression and reported odds-ratios (OR) by setting the control variable to the most commonly reported adverse event, diarrhea (4.62%) and tested the second most common ADR, hyperglycemia (4.43%) and subsequently a rather specific adverse drug reaction such as pneumothorax (3.91%, n=12, male=6, female=6), across the top twenty reported FAERS drugs in our study. We found that risperidone OR=214 (95% confidence interval [CI], 148 – 308, p= 5.60e-183) resulted in the highest odds of causing hyperglycemia and that tacrolimus/tacrolimus anhydrous (n=18, male=11, female=7) OR=1.17 (95% CI, 1.13 – 1.32, p=0.00129) also increased the odds of hyperglycemia. In the preceding analysis we identified methotrexate (n=437, male=196, female=225, undefined=16) OR=0.67 (95% CI, 0.577 – 0.778, p=1.60e-07) increased the odds of diarrhea. Further, we found that ondansetron hydrochloride (n=75, male=22, female=53) OR=7.12 (95% CI, 1.59 – 31.9, p=0.0104) resulted in the highest odds of causing pneumothorax among the top 20 most frequently reported drugs in our study.

**Figure 3.**
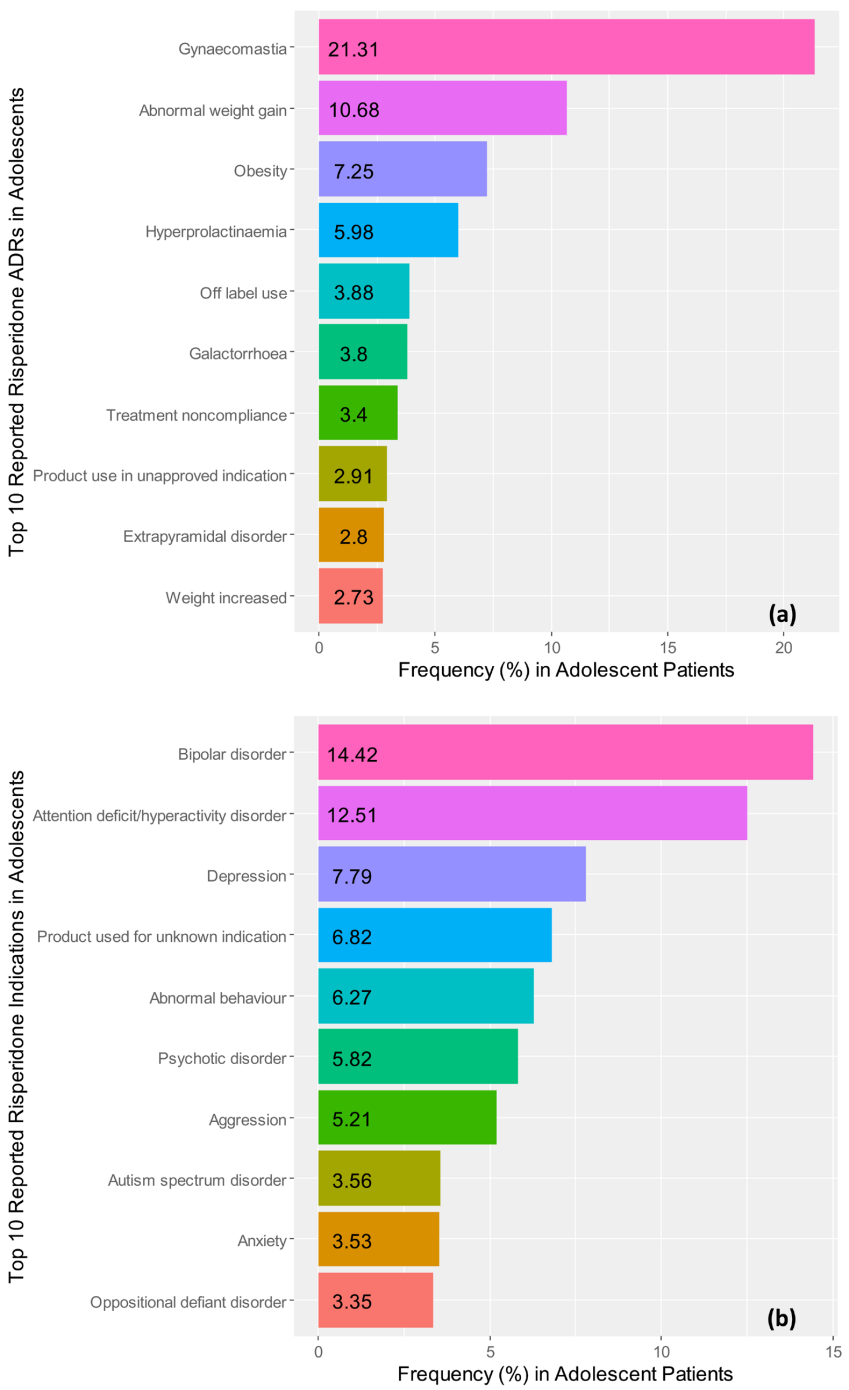
Frequencies for the top 10 reported ( **a**) adverse drug reactions and ( **b**) reported clinical indications for risperidone in adolescent patient records identified in the FDA Adverse Events Reporting system ranging from the 3 ^rd^ quarter of 2014 to the 3 ^rd^ quarter of 2017.

Figure 3a illustrates the top 10 ADR reporting frequencies of risperidone and Figure 3b provides a graphical view of the top 10 clinical indications for prescribing risperidone in the teenage population, as found in our results. The three most frequent ADRs associated with risperidone were reported to be gynecomastia (21.31%), abnormal weight-gain (10.68%), and obesity (7.25%). Further, the top three most frequently reported indications associated with risperidone were reported to be bipolar disorder (14.42%), attention deficit/hyperactivity disorder (12.51%), and depression (7.79%) in teenagers.

**Figure 4.**
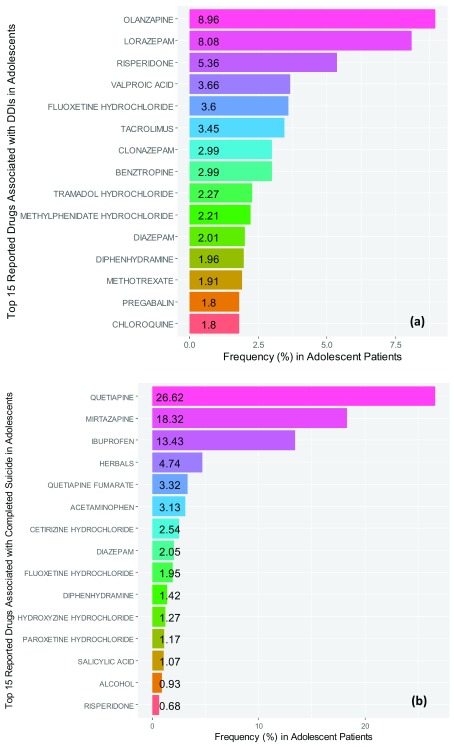
Frequencies for the top 15 reported ( **a**) medications associated with drug-drug interactions (DDIs) and ( **b**) completed suicide in adolescent patient records identified in the FDA Adverse Events Reporting system ranging from the 3 ^rd^ quarter of 2014 to the 3 ^rd^ quarter of 2017.

We identified that the top three medications associated with drug-drug interactions (n=182, male=85, female=97) were olanzapine (8.96%), lorazepam (8.08%), and risperidone (5.36%). The odd-ratios for drugs reported to cause drug-drug interactions were found to be: valproic acid OR=221 (95% CI, 93.90 – 522.00, p=6.20e-35), diazepam OR=170 (95% CI, 62.60 – 463.00, p=7.82e-24), risperidone OR=71.0 (95% CI, 41.40 – 122.00, p=4.17e-54), diphendydramine OR=46.1 (95% CI, 23.60 – 90.00, p=3.56e-29), lorazepam OR=6.08 (95% CI, 4.05 – 9.130, p=3.25e-18), and tacrolimus OR=4.28 (95% CI, 2.73 – 6.71, p=2.45e-10); while amlodipine besylate OR=0.213 (95% CI, 0.1260 – 0.361, p=9.17e-09) was associated with diarrhea in this drug grouping. Figure 4a illustrates the frequencies for the top fifteen reported medications associated with drug-drug interactions.

In assessing the odds-ratios for completed suicide, with a control of diarrhea, among the top twenty associated drugs with completed suicide (n=34, male=8, female=23, undefined=3), we found that ibuprofen OR=188 (95% CI, 105.00 – 335.00, p=4.17e-70) resulted in the highest odds in adolescent cases. Further, we also found, in order of decreasing odds: quetiapine fumarate OR=116 (95% CI, 48.40 – 278.00, p=1.43e-26), diazepam OR=86.0 (95% CI, 32.80 – 225.00, p=1.15e-19), certirizine hydrochloride OR=59.1 (95% CI, 27.90 – 126.00, p=2.33e-26), diphenhydramine OR=16.5 (95% CI, 8.68 – 31.30, p=1.12e-17), and risperidone OR=4.48 (95% CI, 2.27 – 8.82, p=1.49e-05) also were associated with increased odds of completed suicide within adolescent cases.

Contrastingly, hydroxyzine hydrochloride OR=0.0946 (95% CI, 0.0595 – 0.150, p=2.08e-23) and lorazepam OR=0.254 (95% CI, 0.1410 – 0.458, p=5.15e-06) were found to be associated with increased the odds for diarrhea, among top twenty compounds tested for completed suicide. Neither mirtazapine (p=0.980) nor herbals (p=0.990) were associated with increased odds of completed suicide, despite being listed as second and fourth in associated frequency. Similarly, acetaminophen/butalbital (p=0.996), acetaminophen/hydrocodone (p=0.996), alcohol (p=0.996), atorvastatin calcium (p=0.996), carbamazepine (p=0.996), fluoxetine hydrochloride (p=0.993), mirtazapine (p=0.980), paroxetine hydrochloride (p=0.995), and quetiapine (p=0.976, in contrast to quetiapine fumarate p=1.43e-26) did not increase odds of completed suicide, in our analysis. Figure 4b depicts the top fifteen drugs associated with completed suicide in adolescent patient records identified in the FAERS.

The top ten genes associated with ibuprofen, the compound with the highest odds for completed suicide, in this study were found to be *PTGS2* (prostaglandin-endoperoxide synthase 2; 32.79), *PTGS1* (22.74), *ALB* (albumin; 16.90), *CYP2C9* (16.71), *ILB* (interleukin 1 beta; 15.59), *OXAlL* (OXAlL mitochondrial inner membrane protein; 14.28), *IL6* (13.12), *IL10* (12.83), *CYP2C8* (12.64), and *IL1RN* (11.82).

## Discussion

In this study, we chose the adolescent data, over the adult and elderly age groups, in efforts to minimize polypharmacy and to address the scope of the primary aim of our study. We identified pharmacogenes associated with drugs reported with adverse drug reactions and serves as a guide for clinical pharmacology services to prioritize medications in both the inpatient and outpatient care setting. We found that risperidone, a second-generation antipsychotic, with FDA-approval for managing schizophrenia, bipolar I disorder (acute manic/mixed), autistic disorder associated irritability, and Tourette’s syndrome in pediatrics represented the most reported drug in teenagers. We also found that two of the top three most frequently reported indications for risperidone, in adolescent cases, were indications which are not FDA-approved – attention deficit/hyperactivity disorder (12.51%) and depression (7.79%). Thus, these findings highlight the unmet clinical need to increase the number of clinical pharmacology-trained physicians to serve as pharmacogenomic consultants and provide comprehensive patient care advancing genomic medicine ( Boorstein & Historian, 2018).

Prednisolone sodium succinate (3.81%), an anti-inflammatory glucocorticoid with various indications, and the anti-tumor necrosis factor-α (TNF- α) monoclonal antibody – infliximab (3.35%), were second and third in reporting frequency for adolescent patients, respectively. More precise dosing of infliximab may be achieved by pharmacologists using pharmacometrics methods that utilize measured plasma concentrations to recommend doses and dosing intervals to avoid sub-therapeutic concentrations.

To confirm our findings that showed an increased odds of pneumothorax with ondansetron, we used the OpenVigil 2.1-MedDRA (version 2.1, https://www.is.informatik.uni-kiel.de/pvt/OpenVigilMedDRA17/search/) an online pharmacovigilence analysis tool developed by the Christian Albrecht University of Kiel, Germany ( Böhm *et al*., 2016). OpenVigil version 2.1 includes the FAERS data from 4th quarter of 2003 to the first quarter of 2018. Our odansetron-pneumothorax discovery was confirmed by OpenVigil analysis tool and confirmed that for the adolescent age-group ondansetron increased the odds of pneumothorax: Relative Reporting Ratio = 7.037 (95% CI, 2.6 – 19.04), Proportional Reporting Ratio = 7.291 (95% CI, 2.69 – 19.75), and Reporting Odds Ratio = 7.346 (95% CI, 2.69 – 20.06). These results further confirm and strengthen our methodology used in this article. We went a step further and confirmed the increased odds of the ondansetron-pneumothorax association in all age groups using OpenVigil and found: RRR = 5.644 (95% CI, 4.53 – 7.03), PRR = 5.732 (95% CI, 4.6 – 7.14), and OR = 5.751 (95% CI, 4.61 – 7.17).

Therefore, in reference to our results suggesting increased odds of pneumothorax with ondansetron hydrochloride, patients who have a history of pneumothorax, or have conditions with known increased prevalence of pneumothorax (e.g. Marfan’s syndrome, Ehlers-Danlos syndrome, rheumatoid arthritis, poly- and dermato-myositis, ankylosing spondylitis, systemic sclerosis) should be managed with an alternative antiemetic. Further, additional studies should be pursued investigating the mechanisms connective tissue diseases and gene expression modulation with ondansetron.

To understand the pharmacokinetic-pharmacogenomic implications of prescribing risperidone in medical practice, a population pharmacokinetic study reported that relative to normal/extensive CYP2D6 metabolizers, CYP2D6 (*10/*10) poor metabolizers experience a 64% slower oral clearance rate, 72% slower absorption rate in the gastrointestinal tract, and a 53% slower clearance from the central compartment of risperidone to the 9-hydroxyrisperidone metabolite compartment ( Yoo *et al*., 2012). These are striking findings and these same CYP2D6 poor metabolizer’s experience a 3-fold increase in risperidone area-under-the-concentration-time curve (i.e. AUC or drug exposure), when compared to normal metabolizers. To put this into perspective of clinically relevant drug-drug interactions, if a physician prescribes risperidone with either fluoxetine, paroxetine, quinidine, terbinafine, or bupropion (all strong CYP2D6 inhibitors) in a patient who is a normal/extensive CYP2D6 metabolizer, that patient will experience a greater than or equal to 5-fold increase in total risperidone exposure alone, and can be further referenced from the FDA’s Table of Substrates, Inhibitors and Inducers. Moreover, if this patient is has any loss-of-function CYP2D6 single nucleotide polymorphism (SNP), resulting in decreased ability to clear risperidone, the 5-fold increase in total risperidone drug exposure is further increased and could potentially lead to the reported risperidone adverse drug reactions (e.g. gynecomastia, abnormal weight-gain, and obesity). Therefore, caution should be used when associating risperidone with any particular adverse drug reaction alone and should be properly assessed by reviewing the complete medical record by a physician with pharmacogenomics training or clinical pharmacologists.

Approximately nine of the top fifteen reported drugs associated with DDIs, shown in Figure 4a, are prescribed in patients treated for mental health disorders. It may be that patients are experiencing the compounded effects of multiple prescriptions medications competing for the same hepatic biotransformation pathways, coupled with a loss-of-function SNP affecting the primary drug-gene pathway, rather than the later alone ( Storelli *et al.*, 2018). Therefore, these drug-drug-gene interactions resulting in phenoconversion, from a normal metabolizer to a poor or intermediate metabolizer, is an important consultation area for clinical pharmacologists. Similarly, this is another area where the use of TDM and Bayesian dosing support with pharmacometrics may be the most efficient method ( Hiemke *et al.*, 2011; Polasek *et al.*, 2018).

The association between prescription and non-prescription drug pharmacokinetics, drug metabolizing enzymes encoded by cytochrome P450 genes (e.g. CYP2B6, CYP2C19, CYP2D6, etc.) responsible for metabolism of these medicines, and toxicity due to excessively high blood drug levels (i.e. plasma concentrations) resulting in adverse-drug reactions is well established on the FDA’s Table of Pharmacogenomic Biomarkers in Drug Labelling ( https://www.fda.gov/Drugs/ScienceResearch/ucm572698.htm) and the FDA’s Table of Substrates, Inhibitors and Inducers ( https://www.fda.gov/drugs/developmentapprovalprocess/developmentresources/druginteractionslabeling/ucm093664.htm). Further, these known drug-gene interactions, drug-drug-interactions, and extrapolated drug-drug-gene interactions form the basis for the need of pharmacogenomics education within the implementation, education, and practice of Genomic Medicine by physicians, providing consultations by clinical pharmacology trained physicians, and educating various healthcare practitioners to improve safety of prescription and non-prescription medicines. The National Human Genomic Research Institute, of the National Institutes of Health, recently established a Pharmacogenomics Work Group within the Inter-Society Coordinating Committee for Practitioner Education in Genomics (ISCC) to address the need for addressing the pharmacogenomic education needs within clinical genomic medicine ( https://www.genome.gov/27554614/intersociety-coordinating-committee-for-practitioner-education-in-genomics-iscc/). There is a need to increase the number of clinical pharmacology trained physicians in the United States to support the efforts of pharmacogenomics as is already implemented in many countries. Schools of Nursing, Physician Assistant studies, and Pharmacy have already begun integrating the most common drug-gene interactions; however, due to the need for comprehensive medical care, medical students who select training in clinical pharmacology are essential for providing consultations within healthcare systems and in stand-alone clinics.

The limitations and strength of the FDA Adverse Events Reporting System database is that the reports are voluntarily submitted by physicians, pharmacists, lawyers, patient consumers, and various healthcare professionals. Therefore, a limitation is that the complete medical histories are not factored into the analysis and the results are indicative of a subset of all patient adverse drug event experiences. However, despite the limitations, the FAERS database provides insight to the importance of publically available pharmacovigilence data that allows open analysis, discovery for potential repurposing of existing drugs, and provides a reporting mechanism for patients and caregivers to share their medication experiences ( Burkhart *et al.*, 2015; Oshima *et al.*, 2018). Further, we do acknowledge previously published efforts that provide insight to adverse drug reaction reporting in pediatric-aged patients ( Cliff-Eribo *et al*., 2016; De Bie *et al*., 2015; Lee *et al*., 2014).

## Conclusion

In addition to established pharmacogenomic guidelines, the FAERS database provides an important reference point for clinical pharmacologists to use when prioritizing medication safety consultations, pharmacogenomic education, and when seeking to improve hospital outcomes.

## Data availability

The data referenced by this article are under copyright with the following copyright statement: Copyright: © 2018 Eugene AR and Eugene B

Data associated with the article are available under the terms of the Creative Commons Zero "No rights reserved" data waiver (CC0 1.0 Public domain dedication). 

Data used in this study is available from the United States Food and Drug Administration (FDA) website, with specific links provided in Table 1.
