# Supplementary material for: An opportunity for clinical pharmacology trained physicians to improve patient drug safety: A retrospective analysis of adverse drug reactions in teenagers
Source: F1000Res. 2018 Aug 14;7:677. Originally published 2018 May 30. [Version 2] doi: 10.12688/f1000research.14970.2 (PMC6143933; doi:10.12688/f1000research.14970.2)
Supplement: Peer review history

# Peer review: Referee response for version 2

**DOI:** 10.5256/f1000research.17384.r37199

**Published:** 2018-09-14

**Authors:** Daniel D Hawcutt

**Affiliations:** 1 Department of Women's and Children's Health, University of Liverpool, Liverpool, UK

I am happy that the authors have considered the previous points raised, and addressed them.

 This article helps our understanding of the potential harms teenagers can experience from medications.

I have read this submission. I believe that I have an appropriate level of expertise to confirm that it is of an acceptable scientific standard.

# Peer review: Referee response for version 2

**DOI:** 10.5256/f1000research.17384.r37198

**Published:** 2018-08-22

**Authors:** Antonio J. Carcas

**Affiliations:** 1 Clinical Pharmacology Department, IdiPAZ, La Paz University Hospital, School of Medicine, Autonomous University of Madrid, Madrid, Spain

The authors have addressed very satisfactorily the points I raised in my review and I think that has clarified and improved the paper.

I have read this submission. I believe that I have an appropriate level of expertise to confirm that it is of an acceptable scientific standard.

# Peer review: Referee response for version 1

**DOI:** 10.5256/f1000research.16298.r35300

**Published:** 2018-07-16

**Authors:** Daniel D Hawcutt

**Affiliations:** 1 Department of Women's and Children's Health, University of Liverpool, Liverpool, UK

This is an interesting retrospective review of ADR reports submitted to the US FDA for adolescents.

 The strengths are that this population are rarely considered independantly, despite having their own health needs and (as noted by the authors) not having quite the severity of polypharmacy as older individuals. Also, the study considers pharmacogenomics related to the most commonly prescribed medicines, using genecards website as the data source.

 There is a lot of useful data contained in this publication, however there are some aspects that I think could be improved by some clarifications:

 1) What does a genecard score mean for a gene? I know a link is given, but sentence or two giving an overview would help the reader.

 2) I am uncomfortable about the odds ratios (ORs)  for the completed suicide that feature prominently in the results section of the main paper and the abstract (as they are very very large ORs). Are these describing young people who used the drug as the means to commit suicide, or committed suicide while incidentally using this medication (two very different populations). While I appreciated the lack of medical history mentioned in the discussion, I do worry that presenting these finding when compared to diarrhorea may make the drugs look more dangerous than they are (and I have indicated that professional statistical advice would be useful here  to clarify this point - is diarrhoea the right comparitor, or should it be something else?).  

 3) Phrases like "need for precision medicine in psychiatry" in the discussion sound as if they suggest the study has identified new (or overlooked) pharmacogenomic associations that a clinical pharmacologist could act on, but unless I have misunderstood, the study does not do this, it only highlights where areas of unmet pharmacogenomic need exist. The pharmacogenomic section could be removed from the paper and it would still be a good paper, but assuming it is kept, then I think it needs to clearer exactly what information this brings to a clinician.

 Overall, I enjoyed this paper, and it adds to this field.

I have read this submission. I believe that I have an appropriate level of expertise to confirm that it is of an acceptable scientific standard, however I have significant reservations, as outlined above.

# Response

**Published:** 2018-08-08

**Authors:** Andy Eugene

**Affiliations:** Bernard J. Dunn School of Pharmacy, Shenandoah University, USA

Reviewer: 1) What does a genecard score mean for a gene? I know a link is given, but sentence or two giving an overview would help the reader.

**Response: GeneCards uses an Inferred Functionality Score that provides an objective number that indicates the knowledge level about the functionality of human genes relative to the drug queried in the database by comparing the drug with all possible genes. The final results are ranked according to a relevance score and reported in the results section of this article. In our analysis, we mapped the top ten genes using the GeneCards methodology, as has been previously reported ( Stelzer et al., 2016; Weizmann Institute of Science, 2016).**

 Reviewer: 2) I am uncomfortable about the odds ratios (ORs)  for the completed suicide that feature prominently in the results section of the main paper and the abstract (as they are very very large ORs). Are these describing young people who used the drug as the means to commit suicide, or committed suicide while incidentally using this medication (two very different populations). While I appreciated the lack of medical history mentioned in the discussion, I do worry that presenting these finding when compared to diarrhorea may make the drugs look more dangerous than they are (and I have indicated that professional statistical advice would be useful here  to clarify this point - is diarrhoea the right comparitor, or should it be something else?).  

**Response: We thank you for this concern and the current study design with selecting of diarrhea as a control, or comparator, was thoroughly research by examining the frequencies of adverse drug reactions for medications in our exploratory analysis. Please see the updated version of the article for the corroboration of our results for risperidone using the OpenVigil 2.1-MedDRA (version 2.1, https://www.is.informatik.uni-kiel.de/pvt/OpenVigilMedDRA17/search/) an online pharmacovigilence analysis tool developed by the Christian Albrecht University of Kiel, Germany in the discussion section. The reason for the larger odds-ratios is due to the selection of the top 20 drugs ranked and implicated for the adverse drug reaction and not the entire database. Therefore, it is important to note that these are reporting odds-ratios for the top 20 drugs list in the specific adverse drug reaction**.

 Reviewer: 3) Phrases like "need for precision medicine in psychiatry" in the discussion sound as if they suggest the study has identified new (or overlooked) pharmacogenomic associations that a clinical pharmacologist could act on, but unless I have misunderstood, the study does not do this, it only highlights where areas of unmet pharmacogenomic need exist. The pharmacogenomic section could be removed from the paper and it would still be a good paper, but assuming it is kept, then I think it needs to clearer exactly what information this brings to a clinician.

**Response: We thank the referee for the comment and re-wrote the sentence to better reflect the findings and scope of the paper.**

****

**Thus, these findings highlight the unmet clinical need to increase the number of clinical pharmacology-trained physicians to serve as pharmacogenomic consultants and provide comprehensive patient care advancing genomic medicine (Boorstein & Historian, 2018).**

# Peer review: Referee response for version 1

**DOI:** 10.5256/f1000research.16298.r35025

**Published:** 2018-06-26

**Authors:** Antonio J. Carcas

**Affiliations:** 1 Clinical Pharmacology Department, IdiPAZ, La Paz University Hospital, School of Medicine, Autonomous University of Madrid, Madrid, Spain

The aim of this study is to know the most frequently reported drugs associated to adverse drug reactions (and DDIs) in order to prioritize the efforts for clinical pharmacology consultation services. I find this manuscript interesting.

 Comments:

 Just to clarify, please confirm that adolescent age is considered from 12 to 17 yo.

 I agree about the utility of knowing the most frequently reported drugs associated to adverse drug reactions; the author should mention some other similar studies previously published ^1^^-^^4^. I also like the concept that a better knowledge of the most frequent drugs producing AEs can drive prioritization of efforts for clinical pharmacology consultation services. However:

 - Authors should give a more detailed description of the design (case/non-case ?) and statistical methods allowing calculation of OR.

 - We should not disregard the potential of this analysis to rise hypothesis about the link between the AE, DDIs and drug PK and pharmacogenetics. A comment by the authors would be useful.

 - I also think that it would be useful for readers to provide a more specific comment about the relationship between pharmagenomics and AEs whose knowledge could improve drug safety. For example, CYP2D6 polymorphisms have been related to weight gain and hyperprolactinemia in patients with risperidone (including adolescents). On the other side, the association of ondansetron with pneumothorax could be a confounding by indication and not a true causal association.

 Although not new, it is interesting also the finding that frequently reported indications for risperidone were non-approved indications by FDA and that top fifteen reported drugs associated with DDIs are prescribed in patients treated for mental health disorders.

I have read this submission. I believe that I have an appropriate level of expertise to confirm that it is of an acceptable scientific standard, however I have significant reservations, as outlined above.

## References

[1] LeeWJLeeTAPickardASCaskeyRNSchumockGT: Drugs associated with adverse events in children and adolescents. *Pharmacotherapy*.2014;34(9) : 10.1002/phar.1455 918-26 10.1002/phar.1455 24990656

[2] de BieSFerrajoloCStrausSMVerhammeKMBonhoefferJWongICSturkenboomMCGRiP network: Pediatric Drug Safety Surveillance in FDA-AERS: A Description of Adverse Events from GRiP Project. *PLoS One*.2015;10(6) : 10.1371/journal.pone.0130399 e0130399 10.1371/journal.pone.0130399 26090678PMC4474891

[3] RustKRClancyTVWarrenGMertesdorfJMaxwellJG: Mirizzi's syndrome: a contraindication to coelioscopic cholecystectomy. *J Laparoendosc Surg*.1991;1(3) :133-7 175182710.1089/lps.1991.1.133

[4] Cliff-EriboKOSammonsHChoonaraI: Systematic review of paediatric studies of adverse drug reactions from pharmacovigilance databases. *Expert Opin Drug Saf*.2016;15(10) : 10.1080/14740338.2016.1221921 1321-8 10.1080/14740338.2016.1221921 27501085

# Response

**Published:** 2018-08-08

**Authors:** Andy Eugene

**Affiliations:** Bernard J. Dunn School of Pharmacy, Shenandoah University, USA

Reviewer: Just to clarify, please confirm that adolescent age is considered from 12 to 17 yo.

**Response: Yes, in the methods section, I added a comment to specifically reflect the numerical age group for adolescents as (12 – 17 years-old).**

 Reviewer: I agree about the utility of knowing the most frequently reported drugs associated to adverse drug reactions; the author should mention some other similar studies previously published ^1^ ^-^ ^4^. I also like the concept that a better knowledge of the most frequent drugs producing AEs can drive prioritization of efforts for clinical pharmacology consultation services. However:

**Response: We thank you for the recommending the references from similar previously published efforts identifying adverse drug reactions in pediatric-aged patients. Therefore, we added 3 of the four references you recommended in this updated version of our research article.**


**  **

1. **Lee WJ, Lee TA, Pickard AS, Caskey RN, Schumock GT: Drugs associated with adverse events in children and adolescents.Pharmacotherapy. 2014; 34 (9): 918-26 **
2. **de Bie S, Ferrajolo C, Straus SM, Verhamme KM, Bonhoeffer J, Wong IC, Sturkenboom MC, GRiP network: Pediatric Drug Safety Surveillance in FDA-AERS: A Description of Adverse Events from GRiP Project.PLoS One. 2015; 10 (6): e0130399 **
3. **Cliff-Eribo KO, Sammons H, Choonara I: Systematic review of paediatric studies of adverse drug reactions from pharmacovigilance databases.Expert Opin Drug Saf. 2016; 15 (10): 1321-8 **

** **


**So, we added a statement in the article:**

**Further, we do acknowledge previously published efforts that provide insight to adverse drug reaction reporting in pediatric-aged patients (Lee et al. 2014; De Bie et al. 2015; Cliff-Eribo, Sammons, and Choonara 2016).**

 Reviewer: Authors should give a more detailed description of the design (case/non-case ?) and statistical methods allowing calculation of OR.

**Response: We thank the reviewer for this comment and have added three sentences detailing the methodology using in R for calculation of the odds-ratios within the statistics sub-section in the methods of this research manuscript.**

****

**The following expression details the equation for the reporting odds-ratio: OR = (drug-of-interest group with adverse event / control-drug group with adverse event) / (control-drug group with adverse event / control-drug group without an adverse event). The ‘control-drug group with adverse event’ is set to “Diarrhoea” due to that being the most reported adverse drug event and is most commonly reported in FDA reports in the adolescent age population of patients. Morever, as mentioned, the cases (e.g. drug-of-interest group with adverse event) were set to “Hyperglycaemia”, “Pneumothorax”, and “Completed suicide.”**

 Reviewer: We should not disregard the potential of this analysis to rise hypothesis about the link between the AE, DDIs and drug PK and pharmacogenetics. A comment by the authors would be useful.

**Response: The association between prescription and non-prescription drug pharmacokinetics, drug metabolizing enzymes encoded by cytochrome P450 genes (e.g. CYP2B6, CYP2C19, CYP2D6, etc.) responsible for metabolism of these medicines, and toxicity due to excessively high blood drug levels (i.e. plasma concentrations) resulting in adverse-drug reactions is well established on the FDA’s Table of Pharmacogenomic Biomarkers in Drug Labelling ( https://www.fda.gov/Drugs/ScienceResearch/ucm572698.htm) and the FDA’s Table of Substrates, Inhibitors and Inducers ( https://www.fda.gov/drugs/developmentapprovalprocess/developmentresources/druginteractionslabeling/ucm093664.htm). Further, these known drug-gene interactions, drug-drug-interactions, and extrapolated drug-drug-gene interactions form the basis for the need of pharmacogenomics education within the implementation, education, and practice of Genomic Medicine by physicians, providing consultations by clinical pharmacology trained physicians, and educating various healthcare practitioners to improve safety of prescription medicines. The National Human Genomic Research Institute, of the National Institutes of Health, recently established a Pharmacogenomics Work Group within the Inter-Society Coordinating Committee for Practitioner Education in Genomics (ISCC) to address the need for addressing the pharmacogenomic education needs within clinical genomic medicine ( https://www.genome.gov/27554614/intersociety-coordinating-committee-for-practitioner-education-in-genomics-iscc/). There is a need to increase the number of clinical pharmacology trained physicians in the United States to support the efforts of pharmacogenomics as is already implemented in many countries. Schools of Nursing, Physician Assistant studies, and Pharmacy have already begun integrating the most common drug-gene interactions; however, in due to the need for comprehensive medical care, medical students who select training in clinical pharmacology are essential for providing consultations within healthcare systems and in stand-alone clinics.**

 Reviewer: I also think that it would be useful for readers to provide a more specific comment about the relationship between pharmagenomics and AEs whose knowledge could improve drug safety. For example, CYP2D6 polymorphisms have been related to weight gain and hyperprolactinemia in patients with risperidone (including adolescents). On the other side, the association of ondansetron with pneumothorax could be a confounding by indication and not a true causal association.

**Response: To confirm our findings that showed an increased odds of pneumothorax with ondansetron, we used the OpenVigil 2.1-MedDRA (version 2.1, https://www.is.informatik.uni-kiel.de/pvt/OpenVigilMedDRA17/search/) an online pharmacovigilence analysis tool developed by the Christian Albrecht University of Kiel, Germany. OpenVigil version 2.1 includes the FAERS data from 4 ^th^ quarter of 2003 to the first quarter of 2018. The odansetron-pneumothorax association was confirmed using the OpenVigil analysis tool and confirmed that for the reported adolescent age-group cases, ondansetron increased the odds of pneumothorax: Relative Reporting Ratio (RRR) = 7.037 (95% CI, 2.600 – 19.0408), Proportional Reporting Ratio (PRR) = 7.291 (95% CI, 2.6924 – 19.745), and Reporting Odds Ratio (OR) = 7.346 (95% CI, 2.6899 – 20.0613). These results further confirm and strengthen our methodology used in this article. We went a step further and confirmed the increased odds of the ondansetron-pneumothorax association in all age groups using OpenVigil and found: RRR = 5.644 (95% CI, 4.5326 – 7.0289), PRR = 5.732 (95% CI, 4.6030 – 7.1386), and OR = 5.751 (95% CI, 4.6139 – 7.1677).**

****

****

To understand the pharmacokinetic-pharmacogenomic implications of prescribing risperidone in medical practice, a population pharmacokinetic study reported that relative to normal/extensive CYP2D6 metabolizers, CYP2D6 (*10/*10) poor metabolizers experience a 64% slower oral clearance rate, 72% slower absorption rate in the gastrointestinal tract, and a 53% slower clearance from the central compartment of risperidone to 9-hydroxyrisperidone metabolite compartment (Yoo et al. 2012). These are striking findings and these same CYP2D6 poor metabolizer’s experience a 3-fold increase in risperidone in the area under the concentration-time curve (i.e. AUC or drug exposure), when compared to normal metabolizers. To put this into perspective of clinically relevant drug-drug interactions in psychiatry where patients experience unnecessary adverse drug reactions, if a physician prescribes risperidone with either fluoxetine, paroxetine, quinidine, terbinafine, or bupropion (all strong CYP2D6 inhibitors) in a patient who is a normal/extensive CYP2D6 metabolizer, that patient will experience a greater than or equal to 5-fold increase in total risperidone exposure alone, and can be further referenced from the FDA’s Table of Substrates, Inhibitors and Inducers. Moreover, if this patient is has any loss-of-function CYP2D6 genotype, resulting in decreased ability to clear risperidone, the 5-fold increase in total risperidone drug exposure is further increased and could potentially lead to the reported risperidone adverse drug reactions (e.g. gynecomastia, abnormal weight-gain, and obesity). Therefore, caution should be used when associating risperidone withj any particular adverse drug reaction alone and should be properly assessed by reviewing the complete medical record by a physician with pharmacogenomics training or clinical pharmacologists.
